# Supplementary material for: Prognostic utility of submental B/M-Mode ultrasonography for swallowing function assessment in post-stroke pharyngeal dysphagia: a preliminary study
Source: PeerJ. 2025 Sep 22;13:e20046. doi: 10.7717/peerj.20046 (PMC12462686; doi:10.7717/peerj.20046)
Supplement: Supplemental Information 6 [file peerj-13-20046-s006.doc]

**Figure_3___Coordinates_of_the_hyoid_movement_trajectory.xls.** Amend as follows：

**Two-dimensional coordinate system metadata table of the transverse displacement trajectory of the hyoid bone during swallowing.**

**favorable_prognosis_groups.xlsx.** Amend as follows：

**Metadata table of the group with favorable prognosis of dysphagia after stroke.**
